# Traditional chinese medicine for diabetic retinopathy: A systematic review and meta-analysis

**DOI:** 10.1097/MD.0000000000019102

**Published:** 2020-02-14

**Authors:** Bing Pang, Qing-Wei Li, Ya-Li Qin, Guang-Tong Dong, Shuo Feng, Jia Wang, Xiao-Lin Tong, Qing Ni

**Affiliations:** a From Department of Endocrinology, Guang’ anmen Hospital of China Academy of Chinese Medical Sciences, Beijing; b Zhongshan Ophthalmic Center, Sun Yat-sen University, Guangzhou; c Beijing Chinese Medicine Hospital, Capital Medical University; d General Department, Guang’ anmen Hospital of China Academy of Chinese Medical Sciences, Beijing, China.

**Keywords:** diabetic retinopathy, randomized controlled trials, systematic review, traditional chinese medicine

## Abstract

**Background:**

Traditional Chinese medicine (TCM) has been used to treat diabetic complications including diabetic retinopathy for many years.

**Objectives:**

This review was performed to systematically assess the efficacy and safety of TCM for treating non- proliferative diabetic retinopathy (NPDR).

**Methods:**

Retrieval from 7 electronic databases was conducted to determine eligible trials published until March 1, 2018. Randomized controlled trials of NPDR that comparing compound Chinese medicine containing the therapeutic method of activating blood and remove stasis versus controls were included for analysis. Primary outcomes were progression of retinopathy. Secondary outcomes included visual acuity, mean defect of visual field, micro-aneurysms, hemorrhage areas, exudates, capillary nonperfusion areas, hemorheological indicators, oscillatory potentials (Ops), glycated haemoglobin (HbA1c), and adverse events. Data extraction and quality assessment were performed. Results expressing as risk ratios (RRs) or mean differences (MD) were analyzed with a fixed- or random- effect model. *I*^2^ statistics were used to assess heterogeneity.

**Results:**

A total of 33 trials and 3373 participants were included. Findings revealed that no included studies reported the progression of retinopathy. Compared with conventional medicine, TCM was significantly better at improving visual acuity (MD, −0.10; 95% confidence interval [CI] −0.16 to −0.05) and Ops (MD, −4.68, 95% CI −8.51 to −0.85), and reducing the mean defect of visual field (MD, −1.43; 95%CI, −2.17 to −0.68), micro-aneurysms (MD, −4.51; 95% CI, −6.23 to −2.79), hemorrhage areas (MD, −0.62; 95% CI, −1.06 to −0.19), plasma viscosity (MD, −0.10; 95% CI, −0.20 to 0.00), and HbA1c (MD, −0.22; 95% CI, −0.42 to −0.03). Compared with placebo, TCM was also associated with a decline in the number of microaneurysms (MD, −4.35; 95% CI, −6.25 to −2.45), exudates (MD, −0.17; 95% CI −0.31 to −0.03), capillary nonperfusion areas (MD, −0.18; 95% CI, −0.31 to −0.04), and HbA1c (MD, −0.88; 95% CI, −1.44 to −0.32). Compared with blank groups, TCM was superior at decreasing the mean defect of visual field (MD, −0.87; 95% CI −0.95 to −0.79) and the numbers of micro-aneurysms (MD, −3.35; 95% CI, −4.73 to −1.97). Adverse events were also assessed.

**Conclusion:**

Activating blood compound Chinese herbal medicine could help to improve visual acuity, micro-aneurysms and HbA1c. Further trials are needed to provide more reliable evidence.

## 1 Introduction

The dramatic increase in the incidence of diabetes mellitus (DM) is becoming a major public health issue. Parallel with the growing DM pandemic, the occurrence of diabetic retinopathy (DR) is also increasing. DR is the most common cause of preventable blindness in working-aged adults (20–74 years).^[1]^ Epidemiological data from rural China suggested that the incidence was 43% for any retinopathy and 6.3% for vision-threatening retinopathy.^[2]^ Another study of mostly urban Chinese individuals indicated that the prevalence of DR was 8.1% among patients with DM.^[3]^ Vision-threatening retinopathy is serious and irreversible, dramatically affecting the quality of life of diabetic patients. Moreover, the expenses of diabetic vascular complications accounted for 80% of the total direct medical expenses, resulting in a large economic burden for society.^[4]^ Therefore, early prevention and treatment are necessary. However, conventional treatment options are limited and mainly include glucose control, blood pressure and lipid control, aspirin, and lifestyle modifications. No approaches have been developed specifically to prevent and treat DR. More and more other effective measures have been given attention.^[5]^

Recently, traditional Chinese medicine (TCM) has become more popular and drawn more attention due to its positive clinical efficacy.^[6–7]^ Recent clinical and experimental studies have proven that TCM is effective in the prevention and treatment of DR.^[7–10]^ Evidences from the clinical trials has suggested that herbal medicine possibly promotes blood microcirculation, improves vascular endothelial function, protects the blood retinal barrier, and inhibits the oxidation and inflammation state, and so on.^[7,9–10]^ The main basis of treatment in TCM is syndrome differentiation. According to syndrome differentiation, TCM has different treatment principles for DR, such as boosting *qi* and nourishing *yin*, enriching the liver and kidney, invigorating the spleen and removing dampness, and activating blood and removing stasis thus unblocking the collaterals. The use of herbs also differs according to these principles.^[11–12]^ In recent years, there have been many studies that use activating blood herbs for the treatment of DR. According to the TCM theory, blood stasis is 1 of the most important factors in the pathogenesis of DR, and thus activating blood and unblocking the collaterals principle is considered to be the key treatment principle.^[13–14]^ Although there have been some systematic reviews and meta analysis to assess the efficacy and safety of TCM for DR, these studies did not differentiate the categories of the herbs used.^[7,15–16]^ Systematic evidence that summarizing the activating blood compound for DR has been lacking. Therefore, we conducted a systematic review to assess the efficacy and safety of the method of activating blood and removing stasis method for the treatment of DR while taking into account the treatment principles. Our findings should serve as a reference for clinicians seeking effective treatment.

## 2 Method

This review was performed based on the PRISMA statement for reporting of systematic reviews and meta-analysis of health care interventions.^[17]^ The trial registration number is as follows: PROSPERO registration no. CRD42016039367.

### 2.1 Search strategy

We searched the following electronic databases to identify eligible trials published from inception to March 1, 2018: including Cochrane Library, PubMed, EMBASE, Chinese Biomedical Literature Database, Chinese National Knowledge Infrastructure Database, Chinese Science and Technique Journals Database, and the Wanfang Database. Conference abstracts were searched manually. The search terms were as follows: (“diabetic retinopathy” OR “retinal disorders” OR “diabetic eye disease”; “retinal disease” OR “proliferative diabetic retinopathy” OR “diabetic macular edema” OR “diabetic maculopathy” OR “vision loss”) AND (“Chinese herbal medicine” OR “herb” OR “herbal medicine” OR “Chinese herb” OR “traditional Chinese medicine”) AND (“randomized controlled trial” OR “controlled clinical trial” ” OR “clinical trial” OR “clinical research” OR “random” OR “randomly” OR “randomized” OR “control”). Different search strategies were applied for Chinese and foreign language databases. If necessary, we contacted the author of the article for additional data.

### 2.2 Study selection

The inclusion criteria were as follows:

(1) The study included non-proliferative diabetic retinopathy (NPDR) patients who were clearly diagnosed by domestically and internationally recognized criteria;
(2) The study included a randomized controlled trial (RCT);
(3) We assessed use of compound Chinese medicine containing the therapeutic method of activating blood and removing stasis as the treatment group, without restriction for the control group, whether using conventional medicine (CM) (such as Calcium dobesilate, vitamins, etc), placebo, or blank. Basic treatment (glucose control, blood pressure control, and blood lipid regulation) accompanied with both of the groups;
(4) We merely included trials whose treatment duration lasted for 12 weeks or more and whose sample size was more than 30 cases; and
(5) The progression of retinopathy was considered the primary outcome.

The progression of retinopathy refers to the proportion of participants who showed improved progression, or it was not calculated.^[7]^ The secondary outcomes included visual acuity, mean defect of visual field, micro-aneurysm, hemorrhage area, exudate, capillary nonperfusion area, hemorheological indicators (mainly plasma viscosity and high shear blood viscosity), oscillatory potentials (OPs), glycated hemoglobin (HbA1c), as well as adverse events.

The exclusion criteria included the following:

(1) Studies describing interventions combined with other TCM therapies (compound Chinese medicine, traditional Chinese patent medicine, acupuncture or acupoint injection) were excluded;
(2) Non-randomized trials were excluded;
(3) Studies with a treatment duration of less than 12 weeks and/or a sample size of less than 30 cases were excluded.

### 2.3 Data extraction

The details of included trials were extracted independently by 2 authors (Ya-li Qin and Shuo Feng) using a standard data extraction form, which included the following items: general information (title, authors, year published); participant characteristics (sample size, age, gender, duration of DM, and diagnostic criteria); interventions (ingredients and dosage of herbal medicine, details of the control interventions, and duration of treatment); and outcome measures (primary outcome and secondary outcomes). Discrepancies were resolved by consensus or with the involvement of a third party (Qing Ni).

### 2.4 Quality assessment

Two authors (Guang-tong Dong and Jia Wang) assessed the risk of bias in the included studies according to the Cochrane Handbook for Systematic Reviews of Interventions,^[18–19]^ based on 6 items: random sequence generation (selection bias); allocation concealment (selection bias); blinding of participants and personnel (performance bias); blinding of outcome assessment (detection bias); incomplete outcome data (attrition bias); and selective reporting (reporting bias) and other sources of bias. We judged each item from 3 levels: “high risk”, “low risk” and “unclear”, and then we assessed the trials as having a low risk of bias if all items were in the low risk of bias group; a high risk of bias if at least 1 item was in the high risk of bias group; unclear risk of bias if at least 1 item was in unclear. Discrepancies was resolved by consensus or with the involvement of a third party (Xiao-lin Tong).

### 2.5 Statistical analysis

Data regarding outcomes in the eligible trials were combined in the meta-analysis using the Rev Man 5.3 software (Cochrane Collaboration, Oxford). Dichotomous outcomes were indicated as risk ratios (RRs) using the method of Mantel-Haenszel, and continuous variables were indicated as mean differences (MDs) using the method of the inverse variance. All the estimates were calculated as having 95% confidence intervals(CIs). *I*-squared statistics (*I*^2^) were used to assess heterogeneity. A fixed-effect model was adopted if no significant heterogeneity existed (*I*^2^ < 50%); a random-effect model was adopted if significant heterogeneity existed. Publication bias was assessed through funnel plots. Subgroup analysis were performed if the primary outcome demonstrated statistically significant differences between the 2 groups.

## 3 Results

**Figure 1:**
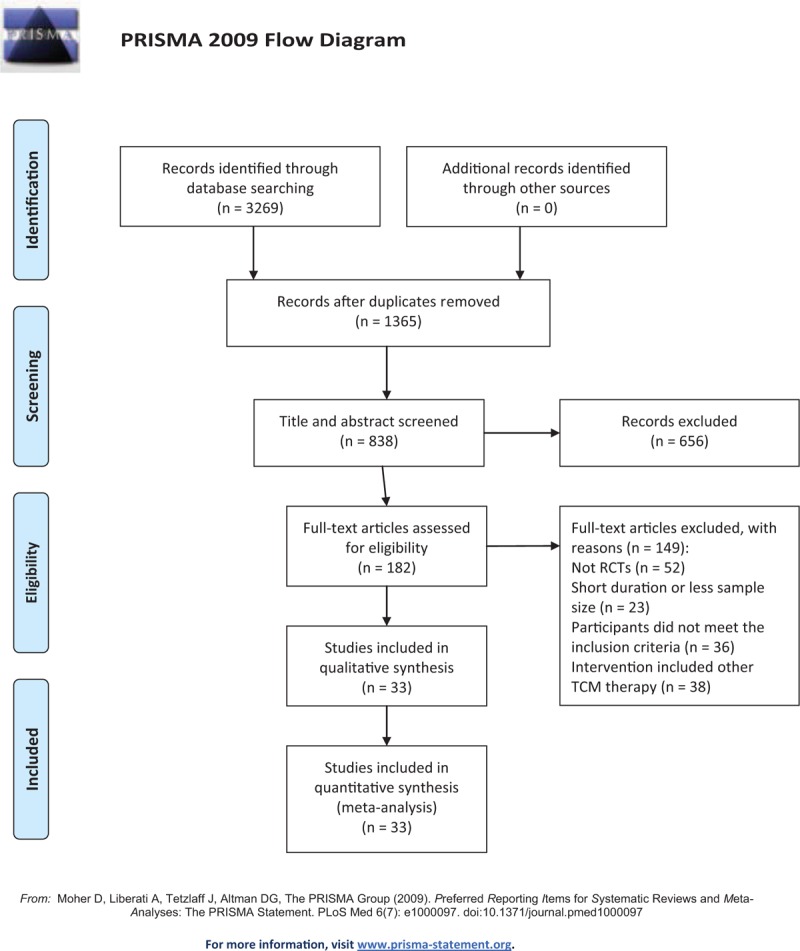
Preferred Reporting Items for Systematic Reviews and Meta-Analyses (PRISMA) flow chart of literature.

Our primary retrieval found 3269 references, and 1904 references were repeated and were excluded. After reading titles and abstracts, the other 656 references were excluded due to repeated literature, experimental studies, retrospective studies, reviews, case reports. This left 182 full texts to be reviewed, and 149 of them were excluded because: they were not RCTs (n = 52), had a short treatment duration or small sample size (n = 23), participants did not meet the inclusion criteria (n = 36), or the intervention included other TCM therapy (n = 38). Finally, 33 RCTs^[20–52]^ were included (Fig. 1).

### 3.1 Description of the included trials

A total of 3430 participants were included (1846 of the intervention group and 1584 of the control group). The sample size ranged from 40 to 360 participants. All the enrolled participants suffered from DM, and most were diagnosed with DR according to diagnostic criteria established in 1985 by the Ophthalmological Society of Chinese Medical Association (OSCMA) or the International Disease Severity Scale for DR, proposed by the Global Diabetic Retinopathy Project Group in 2002. Of the trials, 19 mentioned the syndrome of DR patients according to traditional Chinese medical theory. All were RCTs with 2 parallel arms. In total, 26 trials compared the TCM formula with CM (mainly Calcium Dobesilate), 3 trials compared the TCM formula with a placebo treatment, and 4 trials compared the TCM formula with a blank treatment. Basic treatment (BT) was concomitantly given in both groups to control glycemia. treatment durations varied from 12 to 36 weeks (Table 1  ).

**Table 1:**
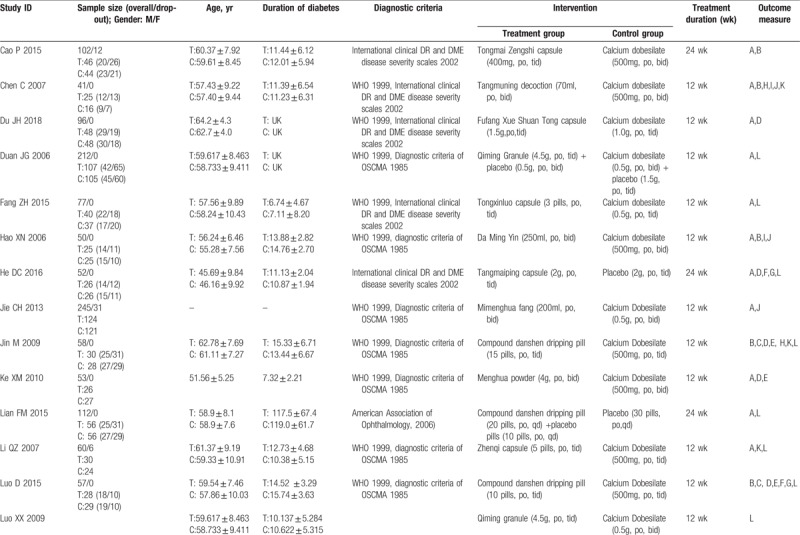
Characteristics of trials included in this review.

**Table 1 (Continued):**
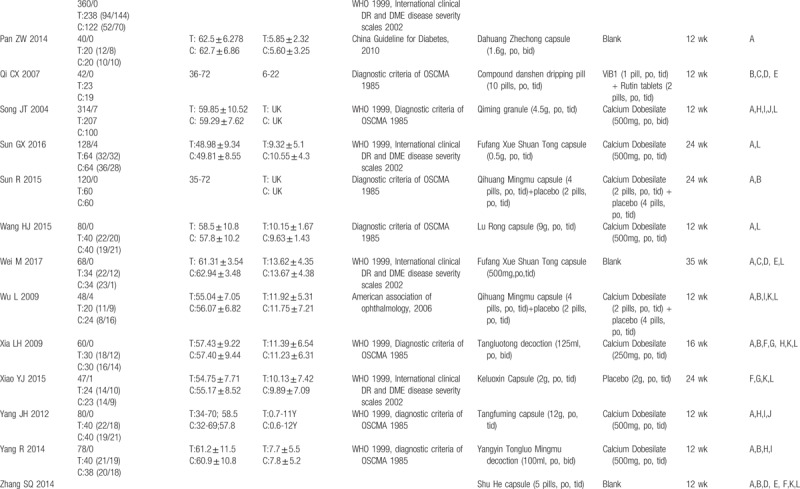
Characteristics of trials included in this review.

**Table 1 (Continued):**
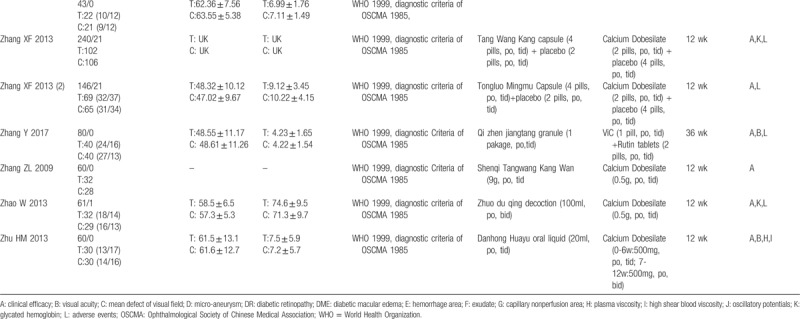
Characteristics of trials included in this review.

### 3.2 Methodological quality

**Figure 2:**
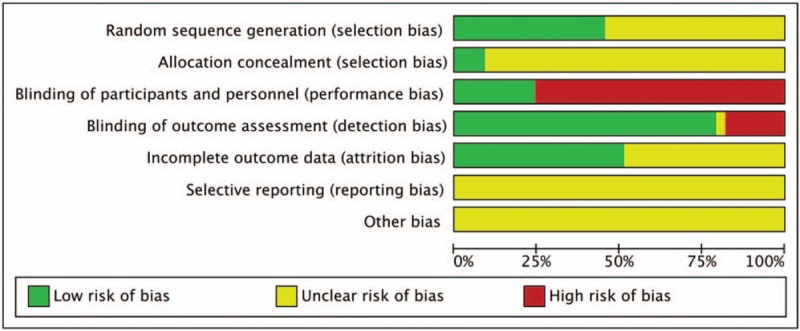
Risk of bias graph.

Fifteen trials described methods of randomization using a random number table or stratified blocked randomization. The remaining trials only indicated “randomly allocating,” with no specific methods of randomization were mentioned. Two trials^[28,41]^ stated how allocation concealment was performed. Eight trials^[20,26,29,38,41,43,47–48]^ used a placebo to conduct the blinding. All trials described the similarities between the intervention and control group. Nine trials^[22,23,33,36,37,39,41,43,47]^ reported dropouts or withdrawals, 3 of whom^[22,37,39]^ reported no drop-out or withdrawal. Selective reporting was difficult to assess, because trial protocols were unavailable (Fig. 2).

### 3.3 Progression of retinopathy

None of the 33 studies reported progression of retinopathy.

### 3.4 Visual acuity

**Figure 3:**
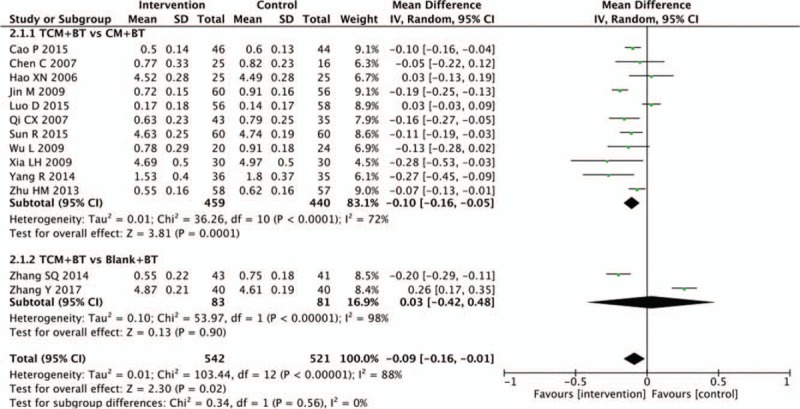
Effects of TCM versus controls on visual acuity. TCM = traditional Chinese medicine.

Thirteen trials reported visual acuity data. A pooled analysis of 11 trials showed a statistically significant increase in visual acuity with TCM, compared to the CM group (n = 899, MD −0.10, 95% CI −0.16 to −0.05, *P* = .0001; *I*^2^ = 72%), while visual acuity differed insignificantly between the TCM and blank groups (n = 164, MD 0.03, 95% CI −0.42 to 0.48, *P* = .90; *I*^2^ = 98%) (Fig. 3).

### 3.5 Mean defect of visual field

**Figure 4:**
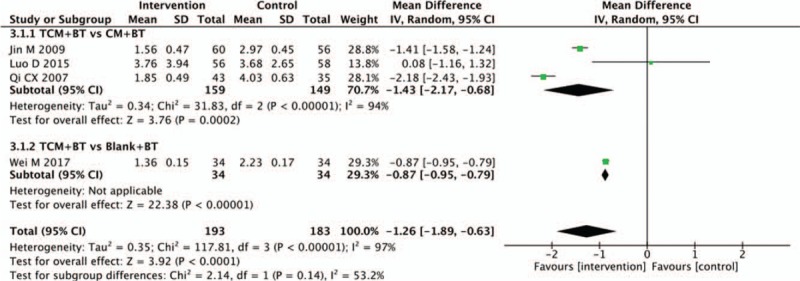
Effects of TCM versus controls on mean defect of visual field. TCM = traditional Chinese medicine.

Four trials provided the improvement of the mean defect of the visual field. Three trials between the TCM and CM groups showed significant differences (n = 308, MD −1.43, 95%CI −2.17 to −0.68, *P* = .0002; *I*^2^ = 94%). In the TCM versus the blank subgroup, only 1 trial reported the mean defect of visual field. There was a significant difference between the 2 groups (n = 68, MD −0.87, 95% CI −0.95 to −0.79, *P* < .00001) (Fig. 4).

### 3.6 Micro-aneurysm

**Figure 5:**
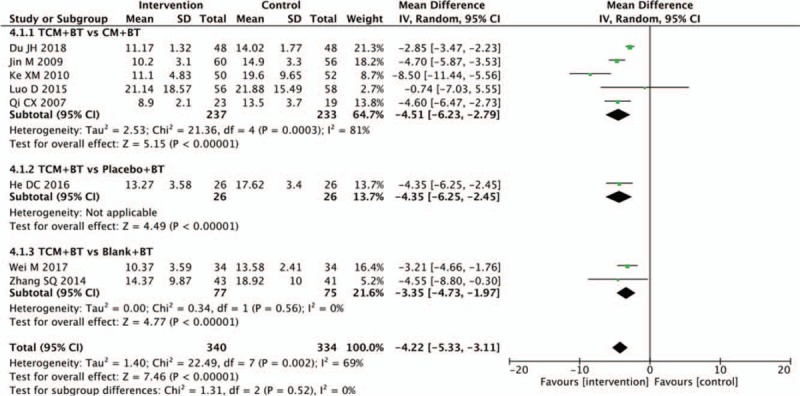
Effects of TCM versus controls on micro-aneurysm. TCM = traditional Chinese medicine.

Eight trials provided the data concerning the number of micro-aneurysms. The number of micro-aneurysms significantly decreased in the TCM group, compared with those in the CM group (n = 470, MD −4.51, 95% CI −6.23 to −2.79, *P* < .00001; *I*^2^ = 81%). There was also a significant difference between the subgroups of the TCM and placebo groups (n = 52, MD −4.35, 95% CI −6.25 to −2.45, *P* <−.00001) and the TCM and blank groups (n = 152, MD −3.35, 95% CI −4.73 to −1.97, *P* <−.00001; *I*^2^ = 0%) (Fig. 5).

### 3.7 Hemorrhage area

**Figure 6:**
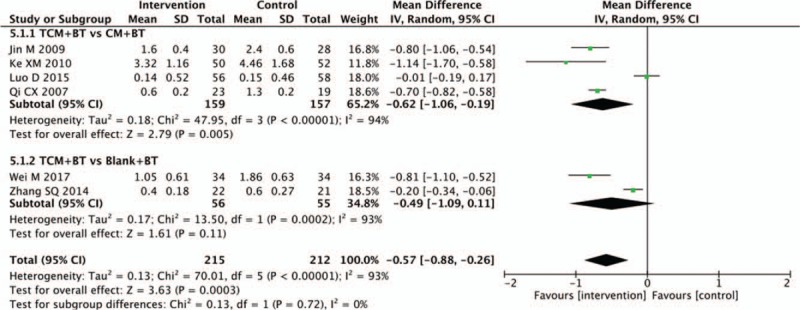
Effects of TCM versus controls on hemorrhage area. TCM = traditional Chinese medicine.

Six trials found the hemorrhage area to be the outcome. The pooled analysis of 4 trials in the subgroup of the TCM versus the CM groups showed a statistically significant reduction in the hemorrhage area (n = 316, MD −0.62, 95% CI −1.06 to −0.19, *P* = .005; *I*^2^ =94%). In 2 trials of TCM, in comparison with blank treatment, results indicated that there was no statistical difference (n = 111, MD −0.49, 95% CI −1.09 to 0.11, *P* = .11; *I*^2^ = 93%) (Fig. 6).

### 3.8 Exudates

**Figure 7:**
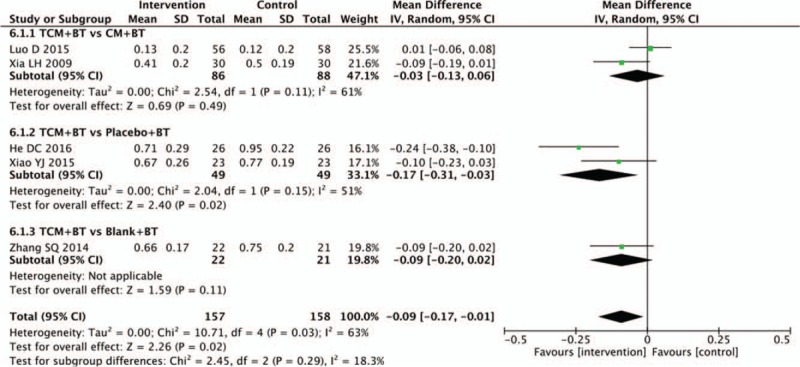
Effects of TCM versus controls on exudates. TCM = traditional Chinese medicine.

In 5 trials, data for the area of retinal exudation were provided. The TCM group was not statistically different than the CM group in decreasing the retinal exudation (n = 174, MD −0.03, 95% CI −0.13 to 0.06, *P* = .49; *I*^2^ = 61%), and no significant difference existed between the TCM and blank groups (1 trial; n = 43, MD −0.09, 95% CI −0.20 to 0.02, *P* = .11). Results showed that there was statistical difference between the TCM and placebo groups in decreasing the exudation area (n = 98, MD −0.17, 95% CI −0.31 to −0.03, *P* = .02; *I*^2^ = 51%) (Fig. 7).

### 3.9 Capillary nonperfusion area

**Figure 8:**
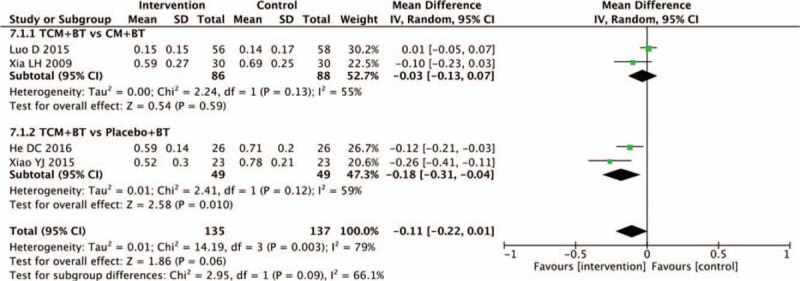
Effects of TCM versus controls on capillary nonperfusion area. TCM = traditional Chinese medicine.

In 4 trials, data for the capillary nonperfusion area were measured. Pooled analysis of 2 trials showed that the TCM group was not statistically different than the control groups in decreasing the capillary nonperfusion area (n = 174, MD −0.03, 95% CI −0.13 to 0.07, *P* = 0.59; *I*^2^ = 55%). However, a significant difference was found between the TCM and placebo groups (n = 98, MD −0.18, 95% CI −0.31 to −0.04, *P* = .010; *I*^2^ = 59%) (Fig. 8).

### 3.10 Hemorheological indicators

**Figure 9:**
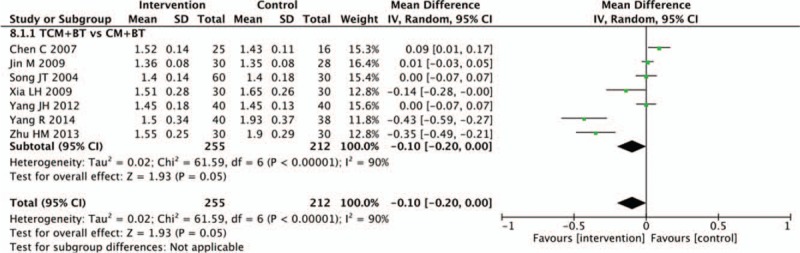
Effects of TCM versus controls on plasma viscosity. TCM = traditional Chinese medicine.

**Figure 10:**
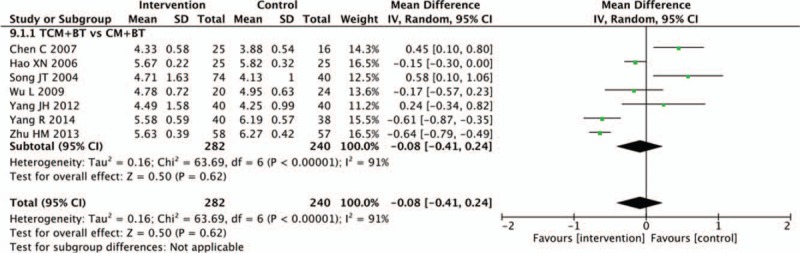
Effects of TCM versus controls on high shear blood viscosity. TCM = traditional Chinese medicine.

Nine trials recorded changes in hemorheology indicators. In this review, we mainly assessed plasma viscosity and high shear blood viscosity. There was no statistical difference between the TCM and CM groups in decreasing plasma viscosity (n = 467, MD −0.10, 95% CI −0.20 to 0.00, *P* = .05; *I*^2^ = 90%). Pooled analysis showed that the TCM group was not statistically different than the CM group in decreasing the high shear blood viscosity (n = 522, MD −0.08, 95% CI −0.41 to 0.24, *P* = .62; *I*^2^ = 91%) (Figs. 9 and 10).

### 3.11 Oscillatory potentials

**Figure 11:**
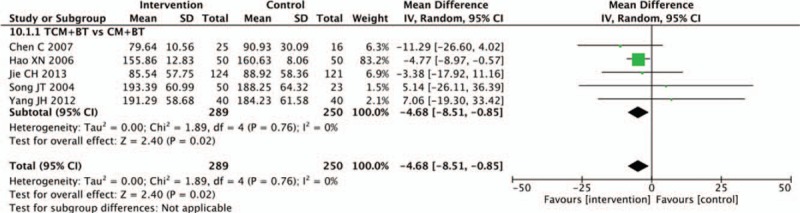
Effects of TCM versus controls on oscillatory potentials. TCM = traditional Chinese medicine.

Five trials compared the effects on oscillatory potentials. Pooled analysis indicated that oscillatory potentials in the TCM group had improved more significantly than in the CM group (n = 539, MD −4.68, 95% CI −8.51 to −0.85, *P* = .02; *I*^2^ = 0%) (Fig. 11).

### 3.12 HbA1C

**Figure 12:**
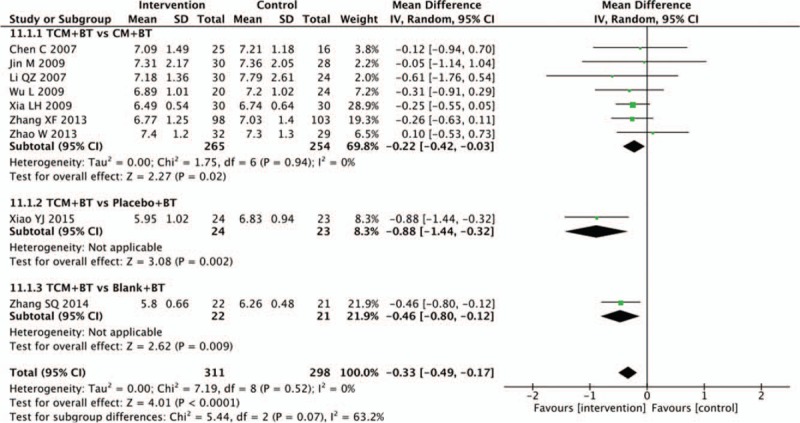
Effects of TCM versus controls on glycated hemoglobin. TCM = traditional Chinese medicine.

Nine trials recorded HbA1c data. Seven trials compared the HbA1c of the TCM to the CM groups, and a meta-analysis demonstrated that participants treated with TCM decreased more significantly than participants receiving CM (n = 519, MD −0.22, 95% CI −0.42 to −0.03, *P* = .02; *I*^2^ = 0%). Results of 1 trial showed that there was a statistical difference between the TCM and placebo groups in decreasing the HbA1c level (n = 47, MD −0.88, 95% CI −1.44 to −0.32, *P* = .002). Results of another trial indicated that there was a statistical difference between the TCM and blank groups in decreasing the HbA1c level (n = 43, MD −0.46, 95% CI −0.80 to −0.12, *P* = .009) (Fig. 12).

### 3.13 Adverse events

**Figure 13:**
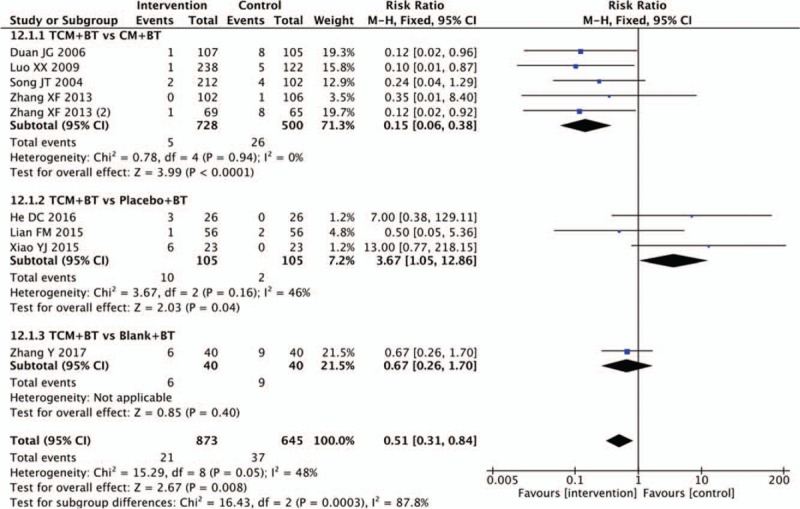
Effects of TCM versus controls on adverse events. TCM = traditional Chinese medicine.

Adverse events (AEs) were reported in 20 trials. Eleven trials^[21,27,31,33,37,39,40,41,42,46,51]^ reported that the TCM groups experienced no AEs, while nine trials recorded the condition of AEs, which were shown in (Fig. 13). Pooled analysis of 5 trials showed that there was a significant difference in the frequency of AEs comparing the TCM with CM group (n = 1228, RR 0.15, 95% CI 0.06 to 0.38, *P* <.0001; *I*^2^ = 0%). Three trials indicated that there was a significant difference in the frequency of AEs (n = 210, RR 3.67, 95% CI 1.05 to 12.86, *P* = 0.04; *I*^2^ = 46%) between the TCM and placebo groups. One trial indicated that there was no significant difference in AEs between the TCM and blank group (n = 80, RR 0.67, 95% CI 0.26 to 1.70, *P* = .40).

Regarding individual AEs, 14 types of AEs were reported in 5 trials that compared TCM with CM. Nausea and vomiting, gastrointestinal fullness, and appetite loss were the 3 most frequently AEs in patients receiving CM. In the subgroup of TCM versus placebo, mild diarrhea was a more frequent AEs in patients receiving TCM. Special information on AEs in Lian FM^[20]^ was unknown. In the subgroup of TCM versus blank, 6 types of AEs were reported, and there was no significant difference between the groups (n = 40, RR 0.73, 95% CI 0.30 to 1.79, *P* = .49) (Table 2).

**Table 2:**
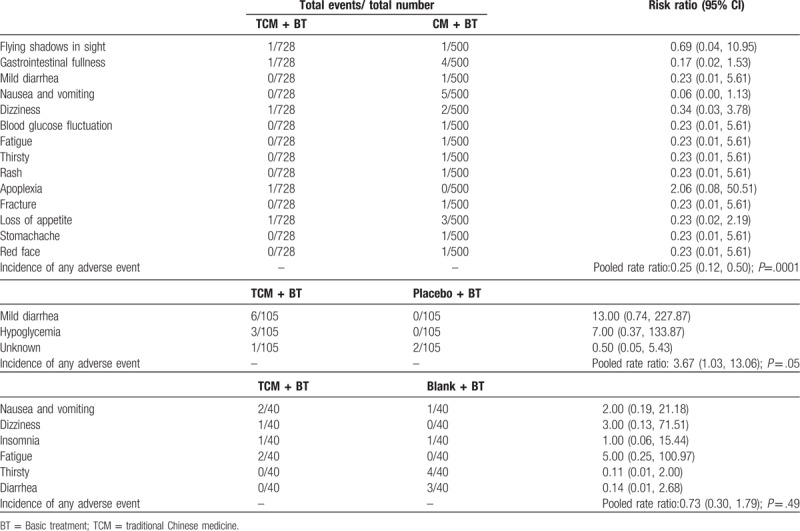
Incidence of adverse events.

### 3.14 Publication bias

**Figure 14:**
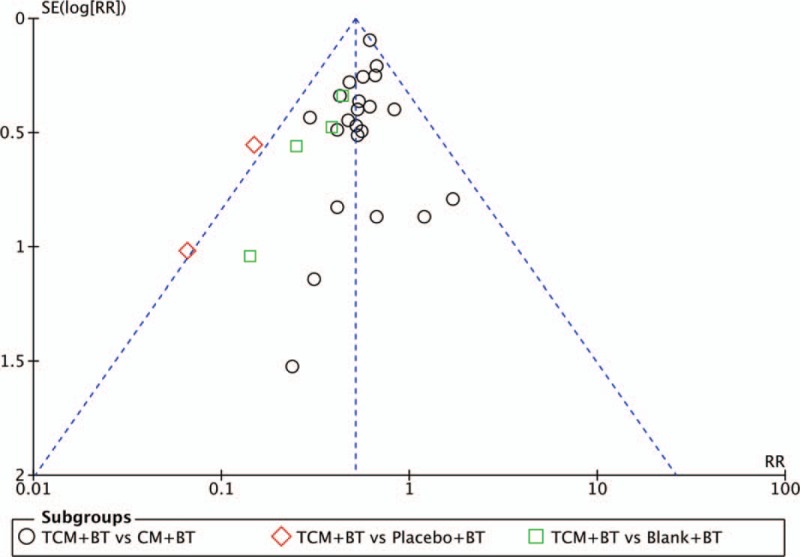
Funnel plot for assessing publication bias.

We performed a funnel plot for clinical efficacy. The funnel shape of the plot was not completely symmetrical, indicating a potential publication bias (Fig. 14).

## 4 Discussion

### 4.1 Summary of evidence

This systematic review enrolled 33 trials involving 3373 participants. The main findings were that, no study reported any data on the progression of DR. Compared with CM, TCM was significantly better at improving visual acuity and oscillatory potentials (OPs), and at reducing the mean defect of visual field, micro-aneurysms, hemorrhage area, plasma viscosity, and HbA1c. Compared with the placebo groups, the interventions of TCM were also associated with a decline in the numbers of micro-aneurysms, exudates, capillary nonperfusion area, and HbA1c. Compared with the blank groups, TCM was superior at decreasing the mean defect of visual field and the numbers of micro-aneurysms. The incidence of AEs in the CM group was higher than that in the TCM group, and nausea and vomiting, gastrointestinal fullness and appetite loss were the 3 most common AEs. Compared with the placebo group, there was a significant difference in the frequency of AEs in the TCM group, and mild diarrhea was the most frequently reported AEs in patients of the TCM group. No statistical significance existed between the TCM and blank groups.

The results suggested participants who took TCM were associated with the increased likelihood of improving visual acuity compared with conventional medication, which was similar to a previous finding.^[7]^ TCM may have been more likely to decrease the number of micro-aneurysms compared to participants who did not take these herbs but used conventional intervention, placebo, or non-treatment. The mechanism may be related to the fact that activating blood herbal medicine possessed the effects of improvement of microcirculation, production of the retinal vascular endothelium, anti-inflammation, anti-oxidation, and so on. In addition, Chinese herbal medicine possessed definite antihyperglycemic effects in decreasing the blood glucose level,^[8,53–54]^ so the meta-analysis results of the HbA1c indicated that TCM was superior to conventional medication, placebo, and non-treatment. From the results of the article, conventional medication had more gastrointestinal side effects, which maybe 1 of the reasons why Chinese patients preferred to choose TCM.

All the included participants were clearly diagnosed with NPDR, but the diagnostic criteria differed, such as the diagnostic criteria of OSCMA established in 1985 and the International Disease Severity Scale for DR proposed by the Global Diabetic Retinopathy Project Group in 2002, and so on. Additionally, 25 types of compound Chinese herbal medicine were included. Although they varied in their herbal components, the formulated prescriptions were based on the principle of “activating the blood circulation and removing stasis”, and formed part of a “group” of herbal medicines with effects of antihyperglycemia, improvement of microcirculation, antiinflammation, and antioxidation designed to decrease blood glucose levels, improve blood rheology, and protect retinal vascular endothelial function.^[8–10]^ The formulations of included TCM contained capsules (16 trials), decoction (6 trials), granules (4 trials), pills (5 trials), powder (1 trials) and oral liquid (1 trial), which were various and highly heterogenous. Regarding the primary outcome, there was a lack of reports on the progression of DR and blinding events. In future research, the primary outcomes should include the occurrence of endpoint events and the progression of DR. The endpoint event was considered as a blinding event. The progression of DR strictly refers to the current international or domestic criteria for the classification or staging of DR, judgement of the progress of DR grading or staging by fundus examination results, and detailed reports on changes in DR grading or staging after treatment. Secondary outcomes mainly focused on laboratory examination and AEs, but the results on assessment of quality of life and disease expenses were rare. The asymmetrical funnel plot demonstrates the potential publication bias. Funnel plots are a visual aid to identify publication bias or systematic heterogeneity. All of the 33 trials were included in these funnel plots, recognizing the heterogeneity of the treatment, trial size, and design. None of the trials found a negative effect, indicating publication bias. Although we undertook extensive searches for unpublished literature, we found no negative trials. However, trials with large positive results are often much easier to publish than trials with negative results. Therefore, it is likely that publication bias is present, affecting the reliability of the meta-analysis.

All the RCTs included in this review were of low quality in terms of design, reporting, and methodology. This provided limited descriptions of study design, randomization and allocation concealment, although all trials stated the randomization procedure they used, only 15 trials provided sufficient information to judge whether randomization was conducted properly and 3 trials stated how allocation was concealed. 8 trials conducted the blinding of participants and personnel, although most of the included trials conducted the blinding of outcome assessment. Half of included trials reported withdrawals or dropouts, and none of the trials mentioned intention-to-treat analysis or had a pre-trial estimation of sample size. Based on the above reasons, the evidences must be interpreted with caution.

### 4.2 Limitation

Several limitations are noteworthy. First, some heterogeneity was found. Although we only included NPDR participants, there was also some heterogeneity in different ingredients, formulations, and dosages of compound Chinese herbal medicine, or different treatment durations across studies, making fully reliable comparisons difficult. Second, regarding the choice of outcomes, the standard of objective assessment is necessary and lacking. Blinding events, the progression of DR, the assessment of quality of life, and disease expenses should be focused on more. Third, the long-term efficacy and safety of TCM on DR are not known. Hence, the pooled results should be treated with caution.

## 5 Conclusion

Preliminary evidence indicated that activating blood compound Chinese herbal medicine may improve the clinical efficacy and may also be associated with the increased likelihood of improving visual acuity and visual function (OPs and mean defect of visual field), compared with conventional medication, and decreased the numbers of micro-aneurysms and HbA1c. However, the methodological quality of trials included in this review were of poor quality. Despite the apparently positive findings, it is premature to conclude the effectiveness of activating blood compound Chinese herbal medicine for the treatment of DR due to the heterogeneity of the included trials and the generally low methodological quality of the included trials. Multi-center, double-blinded, and placebo-controlled RCTs are required to provide stronger evidence.

## Author contributions

**Conceptualization:** Bing Pang, Xiao-lin Tong, Qing Ni.

**Data curation:** Ya-li Qin, Shuo Feng.

**Formal analysis:** Guang-tong Dong, Jia Wang.

**Methodology:** Qing-wei Li.

**Resources:** Xiao-lin Tong, Qing Ni.

**Software:** Shuo Feng, Jia Wang.

**Writing – original draft:** Bing Pang.

**Writing – review and editing:** Bing Pang, Qing-wei Li, Qing Ni.
